# Tolerance to Systemic Isotretinoin Therapy in Two Patients Using Highly Wettable Contact Lenses

**DOI:** 10.1155/2014/452462

**Published:** 2014-01-28

**Authors:** Ayşegül Arman, D. Deniz Demirseren, Gulsen Akoglu

**Affiliations:** ^1^Ophthalmology Clinic, Ankara Atatürk Training and Research Hospital, 06800 Ankara, Turkey; ^2^Dermatology Clinic, Ankara Atatürk Training and Research Hospital, 06800 Ankara, Turkey

## Abstract

*Purpose.* Numerous ocular side effects have been reported with the use of systemic isotretinoin therapy. Herein, we presented two contact lens user patients who did not have contact lens intolerance during systemic isotretinoin therapy. *Methods.* 25-year-old male and 20-year-old female patients with severe acne vulgaris who were using highly wettable silicone hydrogel contact lenses which increase tear film stability were examined. Tear film function tests including Schirmer tests and tear break up time (TBUT) test and ocular surface staining with fluorescein were done. Subjective ocular complaints were scored with ocular surface disease index (OSDI) questionnaire. Patients were followed up monthly and examinations were repeated at each visit. *Results.* Both patients completed the therapy after a cumulative dose of 140 mg/kg isotretinoin in 6 months. The OSDI score and tear film function tests before and after treatment were all within normal limits. *Discussion.* Highly wettable contact lenses that provide increase in the tear film stability may be used during systemic retinoid therapy under close followups. Although isotretinoin affects ocular glands, the differences between tolerances to this retinoid therapy need to be investigated in larger patient groups using contact lenses.

## 1. Introduction

Isotretinoin is a synthetic vitamin A derivative (13-cis retinoic acid) which is widely prescribed for resistant acne vulgaris. Numerous ocular side effects have been reported with the drug affecting the eyelids, cornea, lens, optic nerve, and retina [1]. According to WHO Causality Assessment Guide “ocular sicca, blepharoconjunctivitis, and decreased tolerance to contact lens wear” were reported as “certain” adverse effects among ocular adverse effects of isotretinoin [1]. This report describes two cases who continued to use highly wettable silicone hydrogel contact lenses during systemic isotretinoin therapy without any dry eye complaint, ocular adverse effect, and any need of artificial tears.

## 2. Cases

### 2.1. Clinical Features

Case 1 was a 25-year-old male patient with nodulocystic acne who was consulted from dermatology outpatient clinic before initiation of oral isotretinoin treatment. He has been using contact lenses (Acuvue Oasys with hydraclear plus) for seven years. He was a computer engineer working with computer for about 8 to 10 hours daily.

Case 2 was a 20-year-old female patient with resistant nodulocystic acne who was planned to be put on systemic isotretinoin treatment. She was a student at university. She has been using contact lenses (Acuvue Oasys with hydraclear plus) for three years.

### 2.2. Examinations, Tests, and Followups

Full ophthalmologic examinations were performed. Best corrected visual acuities were detected with the Snellen Chart. Slit lamp examination of anterior chambers and intraocular pressure measurements with Goldmann applanation tonometer were performed. Tear film function tests including Schirmer tests (with and without anesthesia) and tear break up time (TBUT) test and ocular surface staining with fluorescein were done. Subjective ocular complaints were scored with ocular surface disease index (OSDI) questionnaire. Patients were followed monthly and examinations were repeated at each visit.

Before isotretinoin treatment, patients had no ocular complaints regarding dry eye. Best corrected visual acuities were 1.0-1.0 with soft contact lenses. Intraocular pressures and slit lamp examinations of anterior segments were normal. The OSDI score and tear function tests before treatment were all within normal limits (Table 1). Ocular surface staining revealed intact corneal and conjunctival epithelium. Patients stated that they did not want to use glasses instead of contact lenses. Therefore, after informing the patients about possible ocular complications with the use of contact lenses, they were put on therapy with a dosage of 0.8 mg/kg/day oral isotretinoin. Monthly followups were performed by both dermatology and ophthalmology outpatient clinics. Patients continued to use their contact lenses for 8–10 hours daily without any complaint. Their OSDI scores and tear function test results are present in Table 1. Minimal reductions in tear function tests were observed but the results were still within normal limits (Table 1). The patients did not need to use any artificial tears. They completed the therapy with a cumulative dose of 140 mg/kg isotretinoin in 6 months. Additional control examinations one month after the end of systemic treatment revealed normal ocular findings.

## 3. Discussion

Retinoids including vitamin A and synthetic derivatives are used to treat severe resistant nodulocystic acne vulgaris. Among them, oral isotretinoin is the most widely prescribed synthetic vitamin A derivative [1]. The drug causes atrophy of the sebaceous glands and reduces sebum production in the skin. Due to the similarities between sebaceous glands of the skin and meibomian glands of the eye, the drug also causes functional and structural changes in the meibomian glands [2]. Isotretinoin has been shown to be secreted in tears, causing meibomian gland dysfunction. This may result in intolerance to contact lenses, dry eye complaints, and blepharoconjunctivitis [3]. Blepharoconjunctivitis and dry eye are the most common ocular complications and occur in 20–50% of patients in the 3rd to 5th weeks of systemic isotretinoin therapy [4]. On the contrary to all these ocular side effects, our patients had normal ocular surface findings, tear function tests, and no subjective complaint throughout the therapy. They did not experience any dry eye complaints and contact lens intolerance. They were both using silicon hydrogel lenses with 38% water content. Polyvinylpyrrolidone which is embedded within the lens material makes the lens highly wettable and increases the stability of tear film. This feature of the contact lenses might have increased tolerance of both patients to contact lenses throughout the therapy.

Ocular adverse effects of isotretinoin are dose related, being probably the most frequent adverse reactions [5]. The suggested classical dose of isotretinoin is 0.5–1.0 mg/kg/day and the total cumulative dose is usually 120–150 mg/kg within a period of 16 to 30 weeks. The low dose treatment is suggested to be 0.15–0.40 mg/kg and the total cumulative dose is usually less than 120 mg/kg [6]. Although our patients had high dose therapy, they had normal tear function tests and continued to use contact lenses till the end of treatment without the need of artificial tears. We attribute this outcome of therapy to the high levels of tear function tests of the patients before treatment which were preserved by highly wettable contact lenses.

OSDI questionnaire is a reliable method for measuring the severity of dry eye disease [7]. This questionnaire provides a rapid and accurate assessment of the range and severity of dry eye symptoms. OSDI scores of our patients were within normal limits through the therapy and correlated with the TBUT and Schirmer scores.

To the best of our knowledge, there is no study about administration of systemic isotretinoin therapy to contact lens users. Cumurcu et al. reported one case who developed contact lens intolerance in the high dose treatment group [6]. Our report is unique by presenting 2 contact lens users who showed tolerance to systemic isotretinoin therapy without any ocular adverse effect. We suggest that highly wettable contact lenses that provide increase in the tear film stability may be used during systemic retinoid therapy under close followups. Although isotretinoin affects ocular glands, the differences between tolerances to this retinoid therapy need to be investigated in larger patient groups using contact lenses.

## Figures and Tables

**Table 1:** OSDI questionnaire and tear function test results of the patients.

	OSDI		Schirmer without anaesthesia (mm)		Schirmer with anaesthesia		TBUT (seconds)	
Measurement time	Case 1	Case 2	Case 1	Case 2	Case 1	Case 2	Case 1	Case 2

Before treatment	4.17	4.17	35-34	33-33	25-24	23-23	20-20	25-26
At the 2nd month of treatment	4.17	4.17	30-30	27-26	22-22	18-18	15-15	16-16
At the 6th month of treatment	4.17	6.25	23-23	20-19	17-18	14-13	13-13	13-13
One month after completing treatment	4.17	4.17	35-35	35-35	25-25	24-23	20-20	25-25

OSDI: ocular surface disease index; TBUT: tear break up time.
